# X-ray analysis of residual stress gradients in TiN coatings by a Laplace space approach and cross-sectional nanodiffraction: a critical comparison

**DOI:** 10.1107/S0021889813019535

**Published:** 2013-08-24

**Authors:** Mario Stefenelli, Juraj Todt, Angelika Riedl, Werner Ecker, Thomas Müller, Rostislav Daniel, Manfred Burghammer, Jozef Keckes

**Affiliations:** a Materials Center Leoben Forschung GmbH, A-8700 Leoben, Austria; b Department of Materials Physics, Montanuniversität Leoben and Erich Schmid Institute for Materials Science, Austrian Academy of Science, Leoben, Austria; c Rübig GmbH & Co KG, Durisolstrasse 12, A-4600 Wels, Austria; d Department of Physical Metallurgy and Materials Testing, Montanuniversität Leoben, Leoben, Austria; e ESRF, F-38043 Grenoble Cedex 9, France

**Keywords:** residual stress, TiN coatings, Laplace methods, X-ray diffraction, cross-sectional nanodiffraction

## Abstract

Novel scanning synchrotron cross-sectional nanobeam and conventional laboratory as well as synchrotron Laplace X-ray diffraction methods are used to characterize residual stresses in exemplary 11.5 µm-thick TiN coatings. Both real and Laplace space approaches reveal a homogeneous tensile stress state and a very pronounced compressive stress gradient in as-deposited and blasted coatings, respectively. The unique capabilities of the cross-sectional approach operating with a beam size of 100 nm in diameter allow the analysis of stress variation with sub-micrometre resolution at arbitrary depths and the correlation of the stress evolution with the local coating microstructure. Finally, advantages and disadvantages of both approaches are extensively discussed.

## Abstract 2

Residual stresses in as-deposited and blasted TiN coatings are characterized using a Laplace approach and using the novel cross-sectional X-ray nanodiffraction technique. A comparison of real and Laplace space techniques demonstrates the advantages of the nanodiffraction method, with a possibility to analyse local gradients of stress, texture, crystallite size and phase in thin films and coatings.

## 1. Introduction

Protective hard coatings used for high-speed cutting and machining applications in the metal working industry possess complex depth gradients of phases, microstructure and residual stresses. Those gradients can be related to the effects of varying deposition conditions, self-organization phenomena like competitive grain growth, and/or post-deposition mechanical and thermal loads caused, for example, by friction between coating and counterpart (Ohring, 2002 ▶; Cavaleiro & De Hosson, 2006 ▶).

X-ray diffraction (XRD) represents a common technique to evaluate residual stress gradients 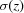 along the surface normal 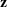 in polycrystalline thin films and coatings from measured X-ray elastic strains using the 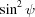 technique (Noyan & Cohen, 1987 ▶; Hauk, 1997 ▶; Birkholz, 2006 ▶; Faurie *et al.*, 2009 ▶). The inclination of the diffraction vector with respect to the sample normal is given by the angle ψ. The measured lattice spacing 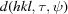 and X-ray elastic strains 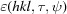 represent volume-average quantities which depend on the actual stress depth profile 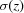, X-ray penetration depth τ, reflection *hkl* and experiment geometry. In general, 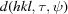 and 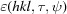 can be related as follows (Dölle & Hauk, 1979 ▶; Genzel, 1996 ▶):
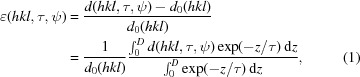
where *D* is the coating thickness and 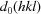 is the unstressed lattice parameter. By varying τ during a diffraction experiment it is possible to evaluate 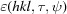 and 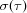 dependencies experimentally in the so-called Laplace space. For the quantification of the stress gradient 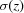 from experimental 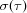 as a function of *z* in real space, however, it is necessary to solve a complex inverse problem similar to inverse Laplace transformation. Since the transformation between 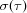 and 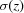 is not ambiguously defined, it is necessary to make very strong assumptions about the actual nature of the 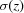 profile in real space (Birkholz, 2006 ▶).

Recently, a novel XRD approach based on cross-sectional nanodiffraction was introduced (Keckes *et al.*, 2012 ▶; Bartosik *et al.*, 2013 ▶). The new technique uses synchrotron point (or pencil) X-ray nanobeams with a diameter (or thickness) down to 100 nm or even less to scan thin films at the cross section in transmission or reflection geometries. The advantage of the new scanning method is that the depth gradients of microstruc­ture, residual stresses and phases can be determined directly in real space as a function of the coating depth *z*. The approach opens the possibility to analyse stresses in graded thin films (Bartosik *et al.*, 2013 ▶) and correlate them with texture, crystallite size and phase gradients. The characteriza­tion of fibre texture is trivial, including also full orien­tation distribution function calculation, especially in the case of in-plane isotropic thin films with a fibre axis oriented perpendicular to the substrate surface (Heidelbach *et al.*, 1999 ▶; Keckes *et al.*, 2012 ▶).

In this work residual stress gradients in exemplary as-deposited and blasted nanocrystalline TiN coatings were analysed (i) using the ‘classical’ Laplace method based on diffraction in reflection geometry and performed in laboratory and synchrotron conditions and (ii) using the recently developed cross-sectional X-ray nanodiffraction approach providing the structural information in real space. The main aim of this work is to compare the two techniques and to discuss their advantages and disadvantages.

## 2. Experimental

### 2.1. Sample preparation

For the depostion of the TiN coating with a final thickness of 11.5 µm on monocrystalline Si(100) substrates, an industrial-sized plasma-assisted chemical vapour deposition and nitriding plant was used. The central component is a hot-wall reactor featuring wall temperatures of up to 873 K. The reactor is supplied with the process gases (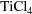, 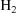, 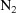, 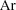) by a standard gas mixing system using mass flow controllers. The plasma is sustained by applying direct current pulses to the substrates. For the deposition, a pressure of 200 Pa was applied. The deposition was carried out in two steps. The first 5 µm of the coating were grown using a substrate temperature of 813 K, and then the temperature was set to 853 K until the final thickness was reached. After the deposition, one coated substrate was blasted using 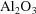 particles with a diameter of 50 µm, applying a pressure of 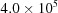
 Pa. The morphology of the as-deposited and blasted coatings was examined using scanning electron microscopy (SEM).

### 2.2. Laboratory monochromatic XRD

**Figure 1:**
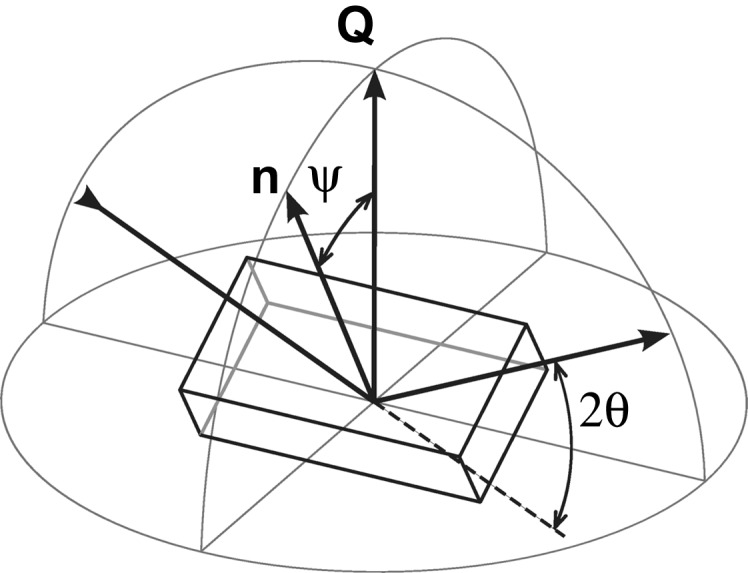
X-ray elastic strains were characterized using the 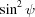 method by measuring lattice spacing 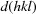 at different sample tilt angles ψ along the direction of the diffraction vector 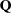. The angle ψ represents the angle between the sample normal 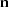 and 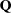. By varying the angle ψ, the X-ray penetration depth τ was tuned (Hauk, 1997 ▶; Birkholz, 2006 ▶).

As-deposited and blasted coatings were characterized using a Rigaku SmartLab five-axis X-ray diffractometer equipped with Cu *K*α radiation, parallel beam optics and a secondary graphite monochromator. The measurements were conducted in side-inclination mode, scanning TiN 200 reflections at different sample tilt angles ψ in the 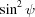 range of 0–0.98 in reflection geometry (Fig. 1 ▶). By tilting the samples around the ψ axis it was possible to vary the X-ray penetration depth τ. The 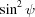 range corresponds to a τ range of approximately 0–1.9 µm, according to (Hauk, 1997 ▶; Birkholz, 2006 ▶)
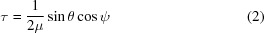
when considering a Bragg angle θ of about 42.6° and a TiN mass attenuation coefficient of 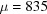
 cm^−1^ (NIST, 2013 ▶) for Cu *K* 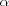 radiation. The lattice spacing 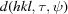 as a function of τ was evaluated with an error smaller than 10%.

### 2.3. Synchrotron energy dispersive XRD

Both TiN samples were characterized also at the Energy Dispersive Diffraction (EDDI) beamline of Helmholtz-Zentrum Berlin (BESSY), Germany (Genzel *et al.*, 2007 ▶). The diffraction experiments were carried out using a white X-ray beam in the energy range 20−100 keV with beam dimensions of 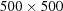
 µm in reflection geometry (Fig. 1 ▶). For the data acquisition, an 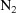-cooled LEGe detector system from Canberry with a resolution of 160 eV at 10 keV and 420 eV at 100 keV was used. The acquisition was performed in a symmetric 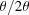 configuration at a constant Bragg angle of 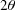 = 12° using a counting time of 60 s per recorded spectrum. The measurements were used to evaluate the lattice spacing 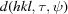 by measuring the positions of TiN 200 reflections at an energy of about 28 keV as a function of the sample tilt angle ψ (Fig. 1 ▶) (Genzel *et al.*, 2004 ▶). The dependence of the lattice spacing 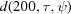 on τ was determined (with an error smaller than 10%) using equation (1) by applying a mass attenuation coefficient of 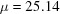
 cm^−1^ (NIST, 2013 ▶).

### 2.4. Cross-sectional synchrotron X-ray nanobeam experiment

**Figure 2:**
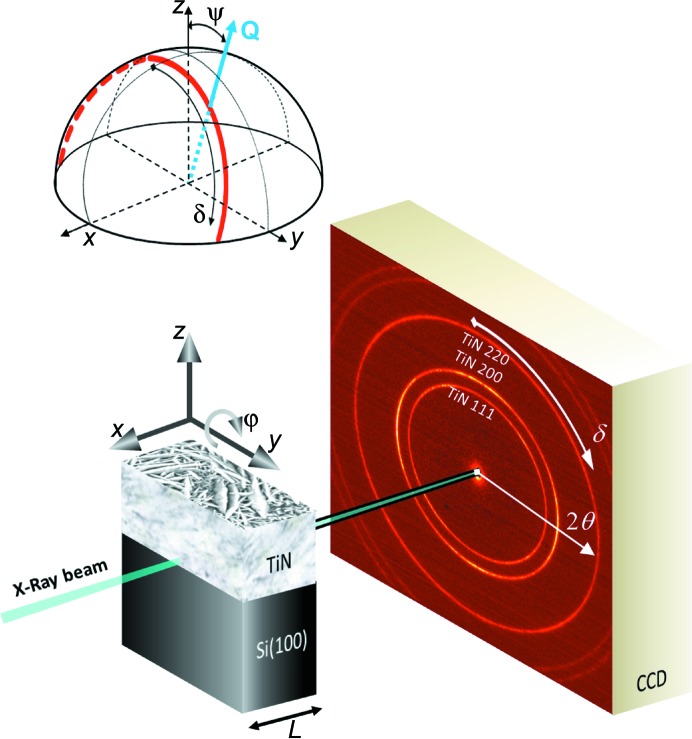
A schematic view of the position-resolved XRD experiment carried out in transmission diffraction geometry using a monochromatic X-ray beam of 100 nm in diameter. The TiN coating on an Si(100) substrate (with a thickness 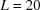
 µm in the beam direction) was moved along the *z* axis with a step size of 100 nm, and diffraction data were collected using a CCD detector at a distance of 102 mm from the sample. The beam was aligned parallel to the sample interface using the ϕ-axis movement. The TiN 111, 200 and 220 Debye–Scherrer rings represent diffraction from TiN crystallites. For a Debye–Scherrer ring, the diffraction vectors 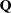 are located on a bold line depicted schematically in the stereographic projection at the top. Note that for 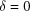, 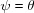.

The strain mapping experiment was conducted at the nanofocus extension of the ID13 beamline of the European Synchrotron Radiation Facility (ESRF) in Grenoble, France (Riekel *et al.*, 2010 ▶). A schematic description of the experimental setup is presented in Fig. 2 ▶. A monochromatic X-ray beam of energy 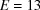
 keV was focused using a Fresnel zone plate (Gorelick *et al.*, 2010 ▶), providing a beam of 100 nm in diameter. Alternatively, using a nanofocusing parabolic refractive X-ray lens (Schroer *et al.*, 2003 ▶), a (pencil-like) beam with dimensions of about 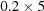
 µm was applied. Both types of setup were tested in order to assess the influence of the beam shape on the diffraction statistics. Owing to the nanocrystalline nature of the coatings, there were, however, no significant differences in the diffraction statistics. In the following only results from experiments performed using a point focus will be presented.

The thin sample slice (with a thickness in the beam direction of 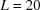
 µm) was aligned with the film/substrate interface oriented parallel to the beam (Fig. 2 ▶) by using the ϕ axis. A CCD area detector with a resolution of 2048 pixels and a pixel size of about 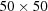
 µm was positioned behind the sample with a sample–detector distance of 102 mm. In order to vertically scan the film cross section, the sample was moved in the beam along the *z* axis with a step width of 100 nm. For each position, the CCD detector acquired a diffraction frame with a counting time of 0.5 s per frame. The two-dimensional diffraction data were processed using the program package *Fit2D* (Hammersley *et al.*, 1996 ▶).

## 3. Results and discussion

### 3.1. Sample surface morphology

**Figure 3:**
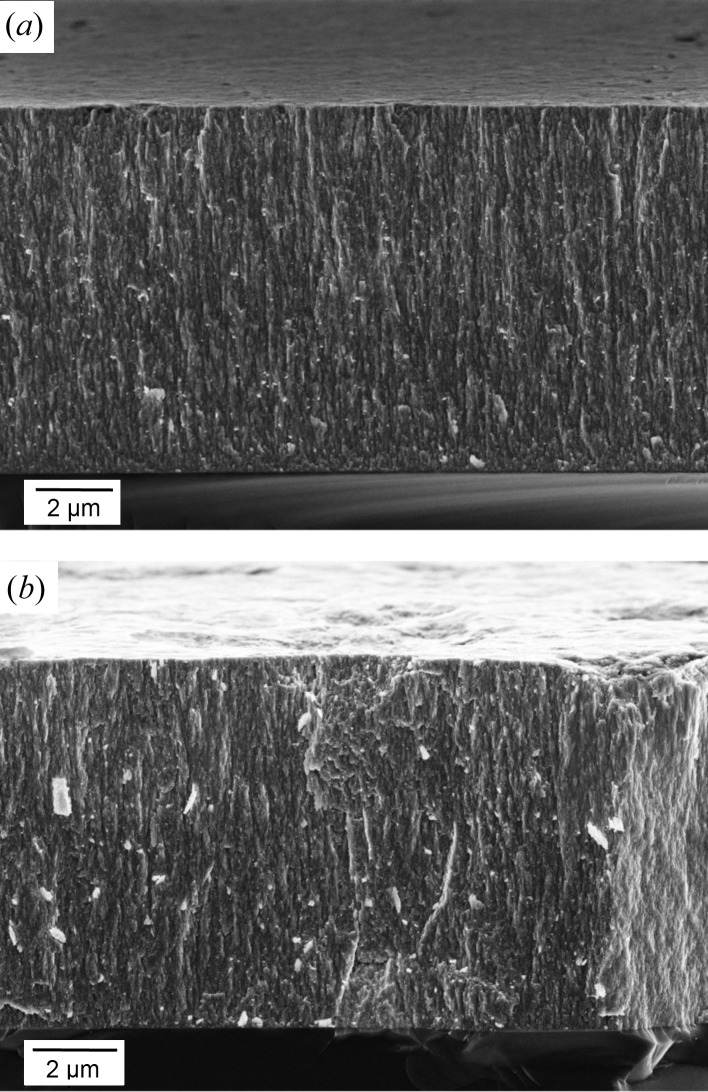
Surface and cross-section morphologies of as-deposited (*a*) and blasted (*b*) TiN hard coatings reveal a nanocrystalline nature of the coatings and an increase of surface roughness of the blasted coating (*b*).

Morphologies and cross sections of as-deposited and blasted TiN coatings were analysed using SEM (*cf.* Fig. 3 ▶). The blasting caused an increase in the surface roughness. The SEM cross sections reveal a nanocrystalline character of the coatings.

### 3.2. Residual stress analysis in Laplace space

For the analysis of residual stresses as a function of coating depth *z* in as-deposited and blasted coatings, it was assumed that the stresses are equibiaxial with 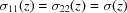 and 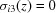 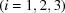 and can be expressed by a parameter 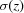. Similarly, for strains, it was supposed that only in-plane 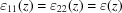 and out-of-plane 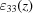 strain components are nonzero (Noyan & Cohen, 1987 ▶; Keckes, 2005 ▶; Renault *et al.*, 2003 ▶).

**Figure 4:**
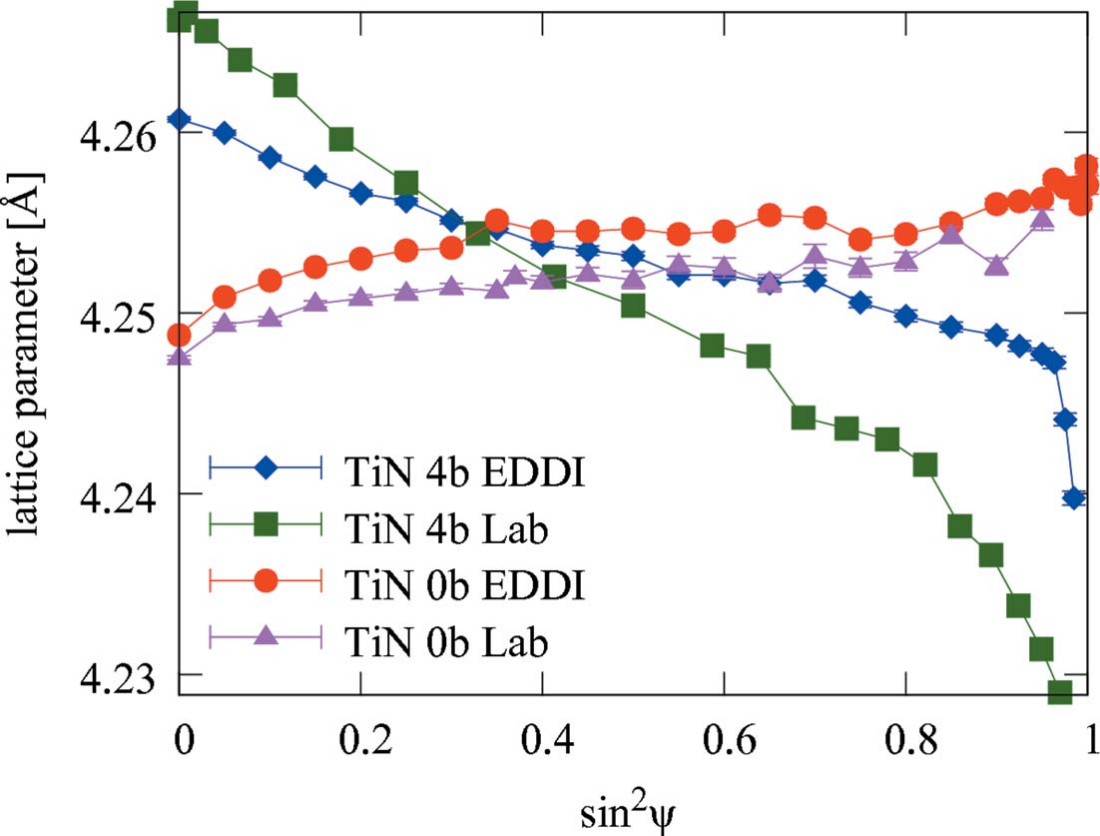
Lattice parameters as a function of 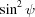 for as-deposited (0b) and blasted (4b) TiN coatings characterized in the laboratory (Lab) and at the EDDI beamline (EDDI). The data were obtained by evaluating the positions of the TiN 200 reflections.

As-deposited and blasted TiN coatings were characterized in the laboratory and at the synchrotron source by analysing the positions of TiN 200 reflections (Fig. 4 ▶). The nearly linear 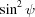 dependencies with positive slopes collected from the as-deposited coating indicate relatively small tensile residual stresses. The different slopes of the dependencies obtained from the as-deposited coating can be interpreted as being caused by the presence of a stress gradient and/or by the different penetration depths of X-rays in the two experiments.

**Figure 5:**
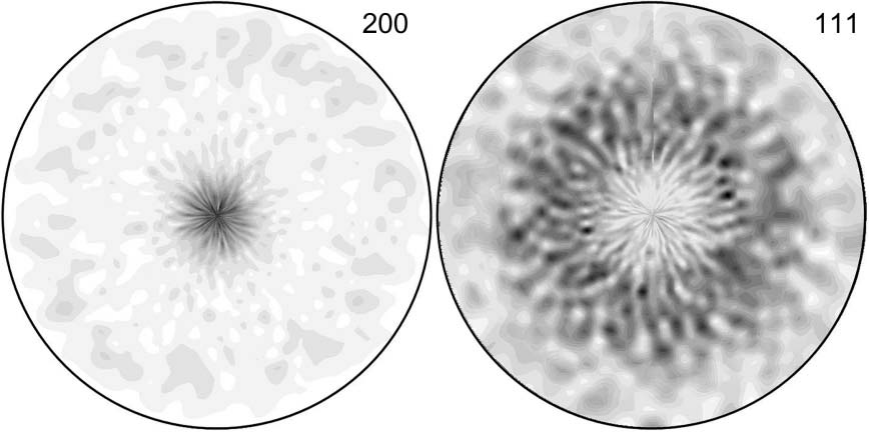
TiN 200 and 111 pole figures collected using a laboratory Rigaku X-ray diffractometer indicate a {100} fibre texture of the TiN coatings.

**Figure 6:**
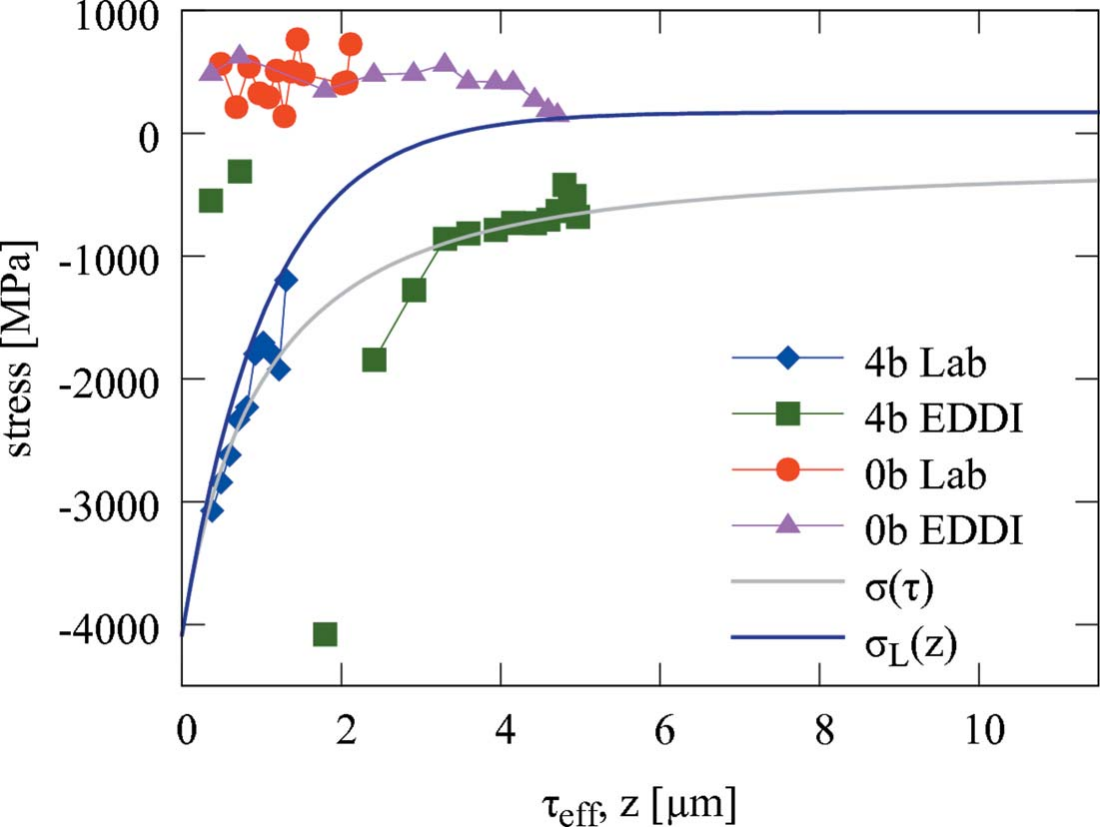
Residual stress values calculated from laboratory (Lab) and synchrotron (EDDI) data (Fig. 4 ▶) using equations (3) [Disp-formula fd4]–(5) for non-blasted (0b) and blasted (4b) coatings. 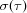 represents the fitted stress profile [equation (7)] in the Laplace space in the blasted coating as a function of 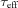. 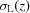 represents the recalculated stress profile [equation (6)] in real space. 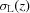 and 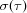 were determined by using both Lab and EDDI data, except for the three EDDI measurement points at 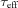 in the range 0–2 µm. For the non-blasted sample the stresses are relatively homogeneous across the coating.

The 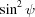 dependencies collected from the blasted coating in both experiments show very pronounced curvatures, which can be interpreted as being caused by the strong gradients of residual stress 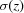 (Hauk, 1997 ▶; Scardi & Dong, 2001 ▶; Birkholz, 2006 ▶). The X-ray elastic strains 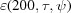 in the coatings were evaluated from 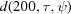 values from Fig. 4 ▶, where the unstressed lattice parameter 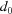 was determined from the intercepts in the 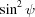 plots. The biaxial rotational symmetric in-plane residual stress in the Laplace space 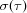 was determined from 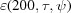 using Hooke’s law as applied in the X-ray diffraction residual stress analysis according to (Genzel, 1997 ▶; Birkholz, 2006 ▶) 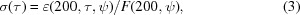
where 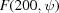 represents X-ray stress factors which depend on the material texture, single-crystal elastic constants, grain interaction, the TiN 200 reflection and the orientation of the diffraction vector (Dölle, 1979 ▶; Houtte & Buyser, 1993 ▶). Since the TiN thin films were in-plane isotropic and possessed a {100} fibre texture (Fig. 5 ▶), the stress factors 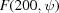 were for simplicity calculated according to (Genzel, 1997 ▶) 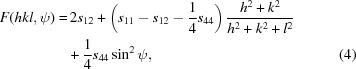
where 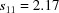, 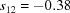, 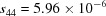
 MPa^−1^ and 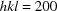 (Kress *et al.*, 1978 ▶). Finally, lattice spacing data obtained from laboratory and synchrotron experiments (Fig. 4 ▶) were used to quantify residual stresses 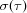 in as-deposited and blasted samples in the Laplace space using equations (2) [Disp-formula fd3]–(4) (Fig. 6 ▶). Since in the case of the synchrotron experiment, performed using relatively hard X-rays, the maximal penetration depth τ exceeds significantly the coating thickness *D*, the ‘information depth’ 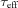 is defined in order to express the origin of the measured information (Hauk, 1997 ▶; Genzel, 1997 ▶):
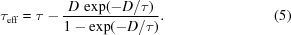
The results from Fig. 6 ▶ document different penetration depths accessible at different facilities. In the case of laboratory measurements performed using Cu *K* 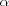 radiation, the maximal penetration depth is about 2 µm, and for synchrotron experiments with much harder X-rays, the maximal information depth 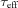 approaches half of the coating thickness (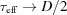) (Genzel, 2005 ▶).

For the as-deposited coating, 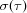 data from Fig. 6  ▶indicate a relatively constant tensile stress state of approximately 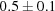
 GPa across the coating. Unfortunately, the stress depth dependence in the interval 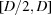 is not accessible (Genzel, 2005 ▶).

In the case of the blasted coating, both laboratory and synchrotron 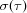 data (Fig. 6 ▶) indicate a decrease in stress with increasing τ. In order to obtain residual stress dependencies 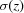 in real space for the blasted coating, it was assumed that the stress depth dependence can be approximately expressed by the exponential function (Hong *et al.*, 2008 ▶)
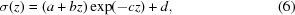
where *a*, *b*, *c* and *d* are numerical constants. The transformation of equation (6) into Laplace space can be expressed as (Genzel, 1997 ▶; Scardi & Dong, 2001 ▶; Birkholz, 2006 ▶) 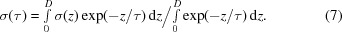
By fitting the numerical parameters *a*, *b*, *c* and *d* from equation (7) to the data of the blasted sample from Fig. 6 ▶, it was possible to evaluate residual stress profiles 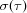 in Laplace space and also 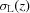 in real space (Fig. 6 ▶). The 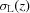 dependence was determined by using both laboratory and synchrotron data (except for the three measurement points from the synchrotron experiment on the blasted sample at τ in the range 0–2 µm). Finally, both 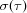 and 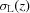 dependencies document an (expected) exponential decrease of the compressive residual stresses as a function of *z* in the blasted TiN.

### 3.3. Residual stress analysis in real space

**Figure 7:**
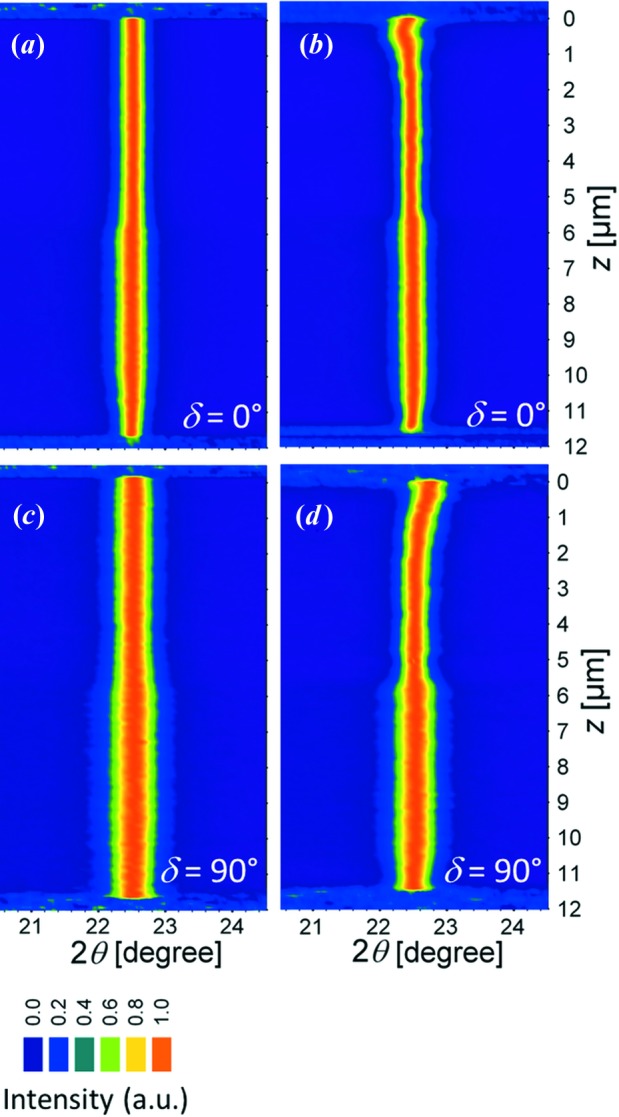
TiN 200 reflections collected at different depths from as-deposited (*a*), (*c*) and blasted (*b*), (*d*) coatings depicted for two diffraction vector orientations δ = 0° (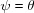) and δ = 90° (*cf.* Fig. 2 ▶). The diffraction peak width varation across the depth is caused (i) by the blasting for depths of 0–2 µm in (*b*), (*d*) and (ii) by the deposition temperature change at about 5.5 µm depth in both samples.

Two-dimensional diffraction patterns obtained from the scanning X-ray nanodiffraction experiment (Fig. 2 ▶) were used to evaluate lattice spacing as a function of the diffraction vector 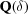 orientation and the coating depth. At first the Debye–Scherrer rings were integrated using the software *Fit2D* in order to analyse the positions of the TiN 200 reflection collected at different azimuthal angles δ. In Fig. 7 ▶, the depth development of the reflection positions 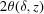 for 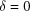 and 90° is presented. In the case of the as-deposited (unblasted) sample, Figs. 7 ▶(*a*) and 7 ▶(*c*) document that the peak positions do not change significantly across the entire coating thickness, which can be interpreted as an absence of a pronounced stress gradient. For the blasted sample, however, the peak positions move towards higher diffraction angles for δ = 90° (Fig. 7 ▶ *d*) and smaller diffraction angles for δ = 0° (Fig. 7 ▶ *b*) for coating depths in the range of about 0–2 µm. This is caused by a pronounced in-plane (near surface) compressive stress which induces a coating contraction in the in-plane direction, that is, a decrease of lattice spacing of crystallographic planes oriented with their normals parallel to the interface. However, the same stress causes an increase of lattice spacing of crystallographic planes oriented with their normals an angle θ with respect to the sample normal. The weak 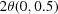 increase (*cf.* Fig. 7 ▶ *b*) and 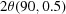 decrease (*cf.* Fig. 7 ▶ *d*) at depths of about 0.5 µm indicate the presence of a weak stress relaxation, which will be discussed further. Different broadening of the TiN 200 reflections for δ = 0° and δ = 90° indicate an anisotropic grain morphology (with needle-like crystallite shapes) and/or anisotropic strains of second and third order (Fig. 7 ▶). The abrupt changes in the peak width at depths of about 5.5 µm were caused by the change in the deposition temperature, which resulted in the growth of larger crystallites with fewer defects at a depth of about 0–5.5 µm.

**Figure 8:**
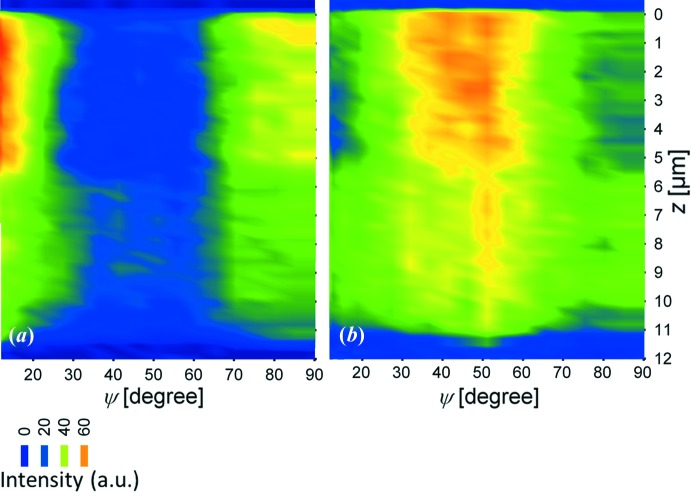
Depth variation of intensities along TiN (*a*) 200 and (*b*) 111 Debye–Scherrer rings in the blasted sample. In agreement with the data from Fig. 5 ▶, the relatively strong intensities at ψ = 10 and 90° for TiN 200 and 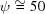
° for TiN 111 document the presence of a {100} fibre texture whose intensity changes as a function of the depth. The increase in the texture sharpness at depths of about 0–5.5 µm was caused by the temperature increase during the deposition.

In order to analyse the depth variation of crystallographic texture in the samples, intensities along TiN Debye–Scherrer rings 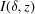 were evaluated. The three dimensional 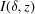 data were transformed into 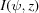 dependencies using a simple transformation from Heidelbach *et al.* (1999 ▶) linking δ and ψ angles (*cf.* Fig. 2 ▶):
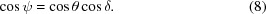
Since the coatings were in-plane isotropic (Fig. 5 ▶), the three-dimensional data collected from the blasted sample (Fig. 8 ▶) indicate the presence of a {100} fibre texture, in agreement with the laboratory measurements from Fig. 5 ▶, where texture intensity changes slightly as a function of the coating depth *z*. In principle, the three-dimensional data can be used to reconstruct the orientation distribution function for every *z* position [as done by Keckes *et al.* (2012 ▶)]; this is, however, out of the scope of this work.

**Figure 9:**
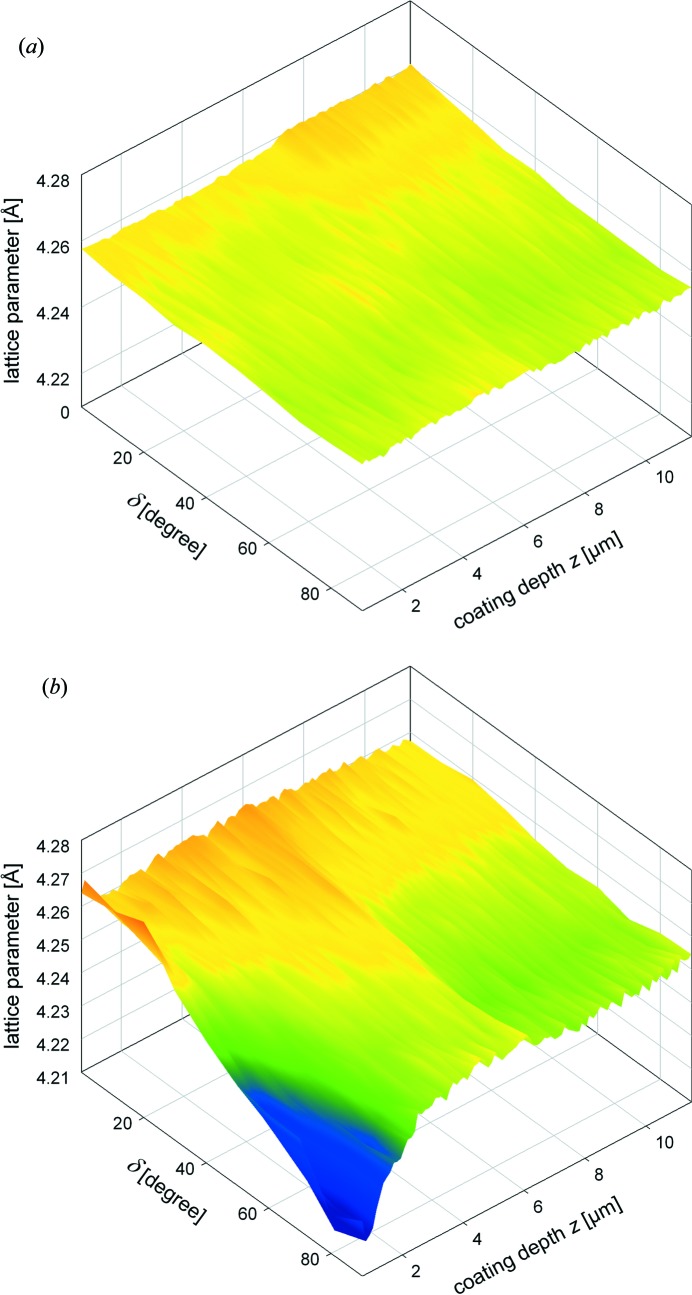
The variations of the lattice spacing 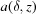 (evaluated from TiN 200 reflections) as a function of the coating depth and the angle δ indicate a relatively homogeneous stress state in the as-deposited coating (*a*) and pronounced residual stresses in the blasted coating surface (*b*).

In order to analyse residual stresses in both coatings, three-dimensional dependencies of the lattice parameter 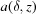 were evaluated from the positions of the TiN 200 reflections (Fig. 9 ▶). The results indicate that, in the as-deposited coating, the lattice parameter does not change significantly as a function of δ at distinct depths *z*. In other words the slope 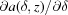 is relatively small. In the case of the blasted coating, however, the slope 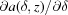 is very pronounced for 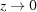, which indicates pronounced stresses in the blasted coating surface.

The three-dimensional 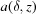 dependencies (Fig. 9 ▶) cannot, however, be automatically used to evaluate residual stresses 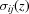 in the coatings. As a result of the sample cutting, the residual stresses in the lamellae [with an intentionally selected small thickness of only 20 µm (Fig. 2 ▶)] were partly relaxed and are not equibiaxial anymore, and therefore 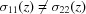 as well as 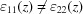. Generally, when for simplicity assuming only a biaxial stress state with negligible 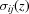, 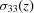 and 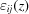, it can be shown that for the measured 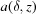 lattice parameters
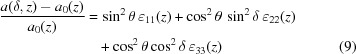
and
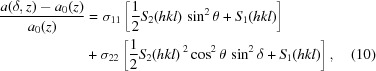
where 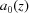 are unstressed lattice parameters. For X-ray elastic constants 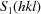 and 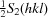, the following equations apply in the case of a {100} fibre texture (Genzel, 1997 ▶):
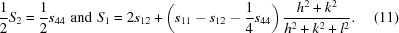
For small Bragg angles θ, the term 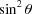 goes to zero, and therefore equations (9) and (10) can be simplified significantly. Moreover equation (10) can be converted as follows:
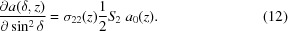
The term 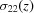 from equation (12) represents in this case the ‘measured’ residual stress component (parallel to the long lamella *y* axis; Fig. 2 ▶), which can be evaluated easily from the distortion of Debye–Scherrer rings expressed by the term 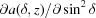. In the case of relatively thick lamellas, where the lamella thickness *L* is a few times the coating thickness *D*, 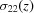 actually represents the depth dependence of the original equibiaxial stress component 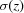, which was present in the coating before cutting. Therefore, 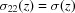 for 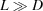. For 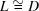, equation (12) can be used to evaluate the original stresses in the coatings, with 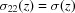 only for samples where the stress relaxation across the coatings is constant, *i.e.* 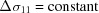. This is the case for coatings and thin films on ductile and soft substrates.

**Figure 10:**
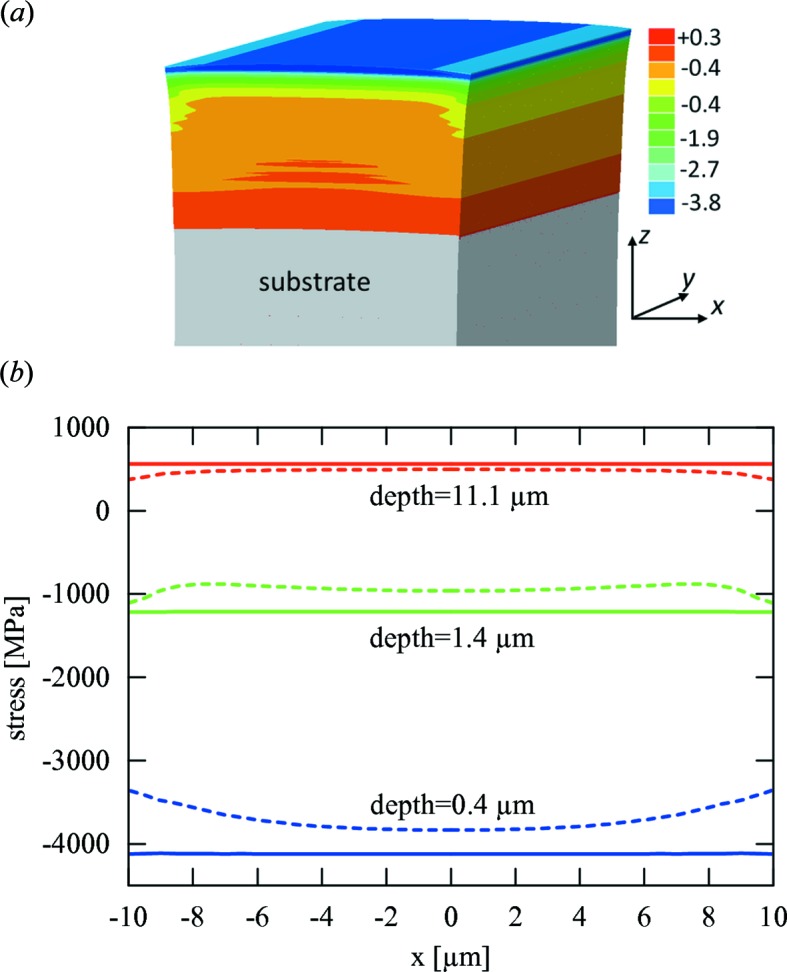
A FEM model documents a relaxation of 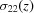 residual stresses (in GPa) in the blasted coating at the lamella cross section due to sample cutting (*a*). In (*b*) solid and dashed lines document the 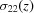 stress magnitude along the *x* axis at different coating depths for the uncut sample and relaxed lamellae, respectively. One can observe that at the interface the relaxation is minimal, while at the surface the relaxation at the lamellae borders is very pronounced.

In the present specific case, of a lamella on a stiff substrate with the thickness *L* comparable to the coating thickness *D* (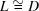), the strain components 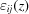 as well as the stress component 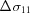 change along the *x* axis at distinct coating depths *z*, owing to the cutting. In other words, the 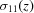 residual stresses relax in a considerable volume fraction of the lamella and cause a depth-dependent strain release. As a result, 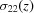 also changes because of this relaxation, especially at the lamella borders (Fig. 10 ▶).

**Figure 11:**
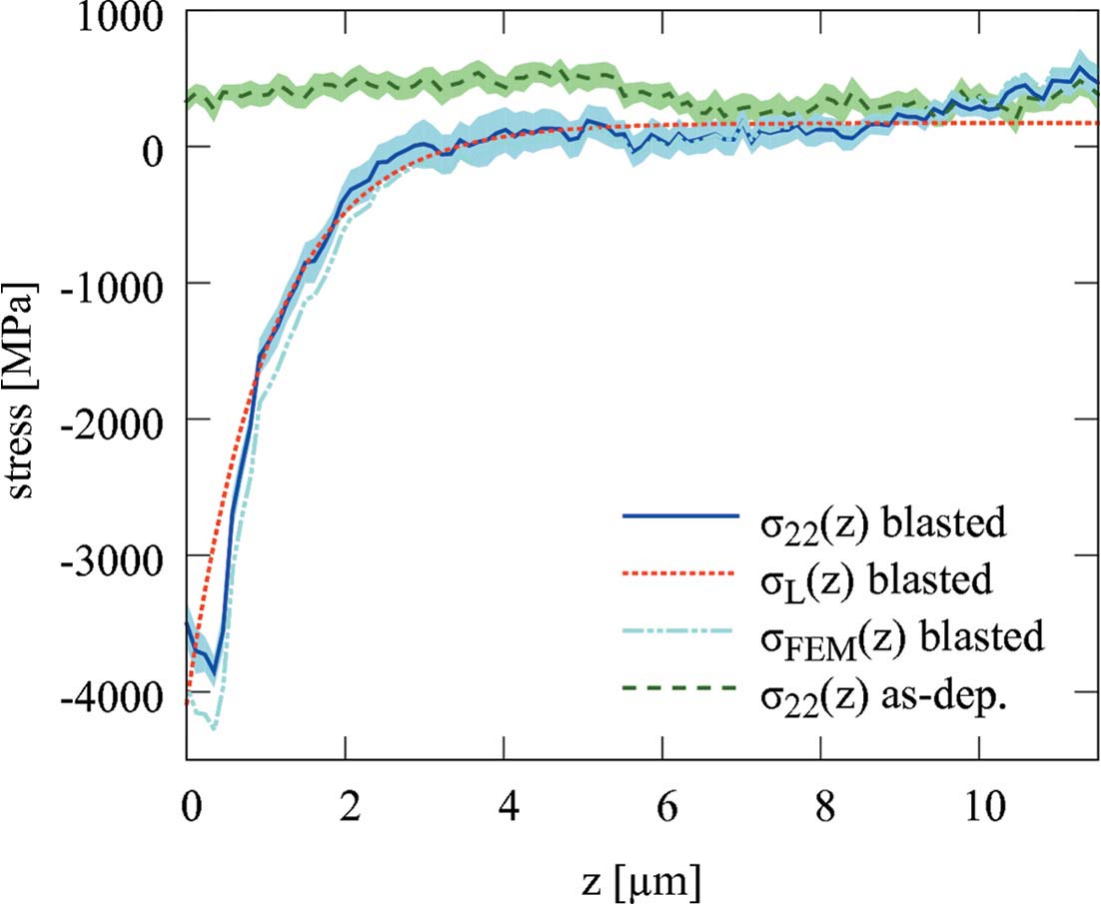
Residual stress gradients in the blasted coating evaluated using the X-ray nanodiffraction approach 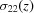 and Laplace method 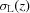. 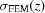 represents the recalculated stress profile using the FEM model. The stresses 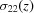 in the as-deposited coating show a small variation across the thickness. The filled bands behind the 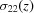 experimental dependencies document the measurement errors. The relatively small tensile stress increase in both samples at the depth of about 5.5 µm was caused by the temperature increase during the deposition.

In order to investigate the effect of the residual stress relaxation due to sample cutting and to reconstruct the original stress state 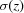 in the blasted sample from 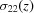 obtained from the nanodiffraction experiment [equation (12)], a finite element model (FEM) was set up in the software package *ABAQUS* (http://en.wikipedia.org/wiki/Abaqus). This model, in which the equibiaxial residual stress profile was applied to the coating as a predefined field, consisted of two steps. In the first step, the boundary conditions were chosen such that the coated sample behaved as if it had an infinite extension in the **x** and **y** directions. The sample was allowed to expand and curve in order to find its mechanical equilibrium. In the second step, the boundary conditions were modified so that a sample with 20 µm thickness in the **x** direction, like in the nanobeam experiment, was formed. In a first simulation, the stress profile obtained from the nanobeam experiment was applied to the uncut sample, and the relaxation after the cut was simulated. This gave a first impression of the relaxation taking place when the lamella is cut. Subsequently, the simulation was integrated in an iterative optimization procedure, altering the stress state applied as the predefined field until the stress state in the 20 µm-thick lamella matched with 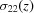 obtained from the nanobeam experiment. Finally, the corrected biaxial stress distribution 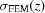 was obtained from the simulation step modelling the uncut sample. The mechanical behaviour of the TiN coating was modelled using values of Young’s modulus and Poisson’s number of 428 GPa and 0.2 from Kress *et al.* (1978 ▶). In Fig. 11 ▶, the evaluated residual stresses 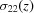 obtained from the X-ray nanobeam experiment, the stresses obtained from the FEM procedure 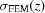 and the stresses obtained from the Laplace technique 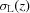 (Fig. 6 ▶) are presented for the blasted coating. Qualitatively, 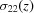, 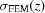 and 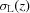 indicate a relatively large magnitude of compressive residual stresses in the blasted coating near the surface and an exponential stress decrease towards the substrate. The comparison of 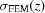 and 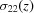 documents the intensity of the stress relaxation, which is significant especially in the coating surface, in agreement with Fig. 10 ▶. Remarkably, the nanodiffraction data indicate a compressive stress maximum at *z* = 0.5 µm. Its presence is also visible in the raw data from Figs. 7 ▶ and 9 ▶. This type of stress gradient has also been reported from simulations of the shot peening process (Hong *et al.*, 2008 ▶). For the as-deposited coating, the stresses are tensile and relatively constant at about 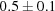
 GPa.

In the 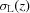 dependence, such local stress variation obviously cannot be resolved, which is a result of the form of equation (6) as well as the principle of the method itself. The remarkable scattering of the first three points in the τ range of 0–2 µm obtained from the synchrotron experiment on the blasted sample (Fig. 6 ▶), however, indicates some irregular stress–depth behaviour, which could be caused by the compressive stress maximum at *z* = 0.5 µm (Fig. 11 ▶).

## 4. Discussion

The aim of this section is to discuss advantages and disadvantages of the Laplace and cross-sectional XRD approaches. Intentionally, a coating with a relatively simple stress depth profile was chosen in order to compare the two approaches.

The main advantages of the Laplace approach are obviously the facts that no sample preparation is required and that it is possible to perform the experiments in the laboratory. The main restriction of the Laplace technique is the necessity to perform an inverse Laplace transformation of the measured 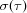 profile. For the transformation, the functional dependence of the residual stress in real space 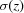 must be pre-selected. In general, there are infinitely many 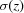 profiles in real space that would result in the same 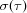 dependence in the Laplace space and *vice versa*. Moreover the often unknown depth dependences of the unstressed lattice parameter 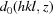 and/or mass attenuation coefficient μ, caused for example by chemical gradients, coating grain morphology and/or density variations, present also a serious problem for the 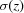 recalculation. The experimental determination of 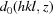 in nanocrystalline and/or graded thin films and coatings from X-ray elastic constants is very questionable. Practically, the Laplace technique can be used to analyse only relatively simple (monotonic) stress profiles 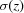 and is not sensitive to local stress variation. Given the nature of the method, an analysis of an oscillating or step-like stress field (Keckes *et al.*, 2012 ▶) is practically impossible.

In the case of the cross-sectional nanobeam approach, no assumptions about the existing stress profile have to be made. This novel technique allows the characterization of very complex stress gradients with step-like or even oscillating depth profiles. Moreover, this powerful approach can be used to evaluate for the first time not only stress but also representative microstructure (texture, crystallite size and defect density) and phase gradients in thin films and coatings. In this way, also the origins of stress evolution across the thickness can be studied and correlated with the deposition conditions (as in the present case) or with the thin-film thermal and/or loading history. It can be expected that this novel approach will allow the development of depth-dependent grain interaction models for thin films and coatings, whose application will be necessary in order to evaluate stresses in samples with strong texture gradients (Keckes *et al.*, 2012 ▶). The main disadvantages of the new approach are the need for sample preparation, a synchrotron beamline providing an X-ray nanobeam and a relatively extensive data treatment, and the necessity to use a FEM model to recalculate the original stresses from the measured data in the case of thin lamellae structures, as in the present case. Currently, however, it is not necessary to perform the residual stress characterization only on thin lamellae with 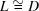. High-energy synchrotron X-ray beams and their high brilliance allow the measurements to be performed also on lamellae with a thickness *L* in the range of 100 µm or even more where the stress relaxation on both sides of the lamella (Fig. 10 ▶) is practically negligible.

In future it can be expected that novel developments in focusing X-ray optics and beamline instrumentation with beam sizes in the sub-100 nm range will result in the possibility of performing even more local characterization of stress gradients. In that case, the application of pencil X-ray nanobeams for coatings and thin films with plain interfaces (Krywka *et al.*, 2012 ▶, 2013 ▶) will rapidly increase in importance in order to guarantee sufficient diffraction statistics, especially when looking for representative microstructure data.
